# Mucoid vasculopathy complicated by multiple giant pseudoaneurysms following Bentall procedure

**DOI:** 10.1186/s44348-024-00011-8

**Published:** 2024-06-12

**Authors:** Sudipta Mondal, Jayakrishnan Radhakrishnan, Bijulal Sasidharan, Vivek V. Pillai

**Affiliations:** 1 Department of Cardiology, Sree Chitra Tirunal Institute for Medical Sciences and Technology, Jai Nagar W Rd, Thiruvananthapuram, Kerala 695011 India; 2 Department of Imaging Sciences & Intervention Radiology, Sree Chitra Tirunal Institute for Medical Sciences and Technology, Thiruvananthapuram, India; 3 Department of CVTS, Sree Chitra Tirunal Institute for Medical Sciences and Technology, Thiruvananthapuram, India

**Keywords:** Mucoid vasculopathy, Pseudoaneurysm, Bentall, Giant, Case report

**Fig. 1:**
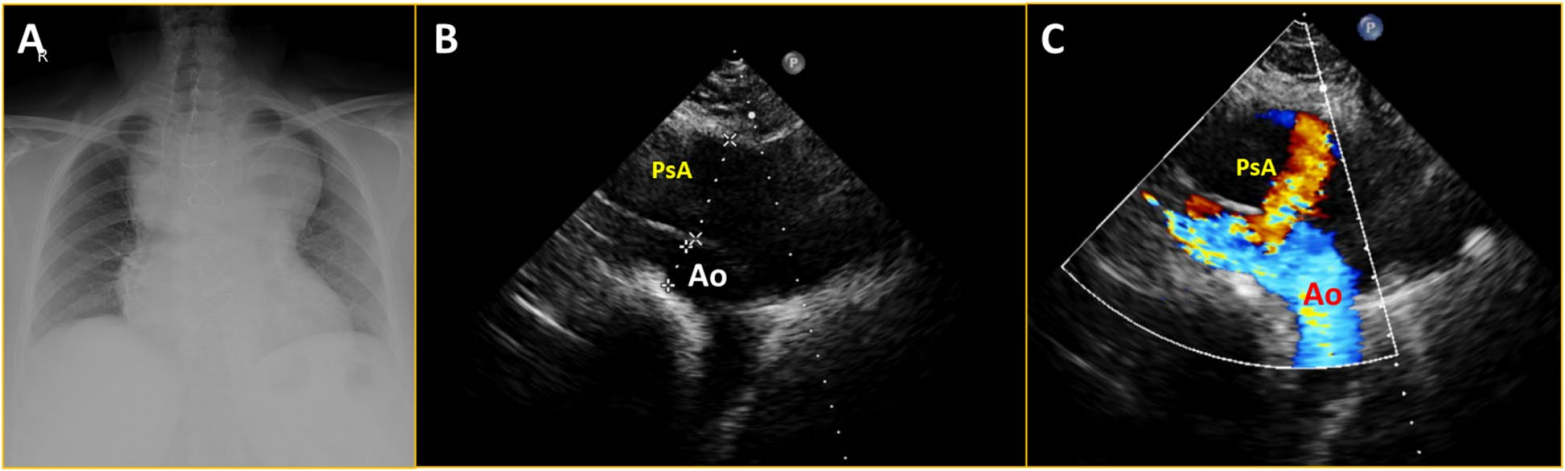
Chest X-ray and 2D echocardiogram. **A** Chest X-ray showing huge mediastinal mass. **B**, **C** Modified parasternal view showing huge pseudoaneurysm anterior to aorta with discrete neck of the pseudoaneurysm with turbulent flow within. PsA = pseudoaneurysm; Ao = aorta

**Fig. 2:**
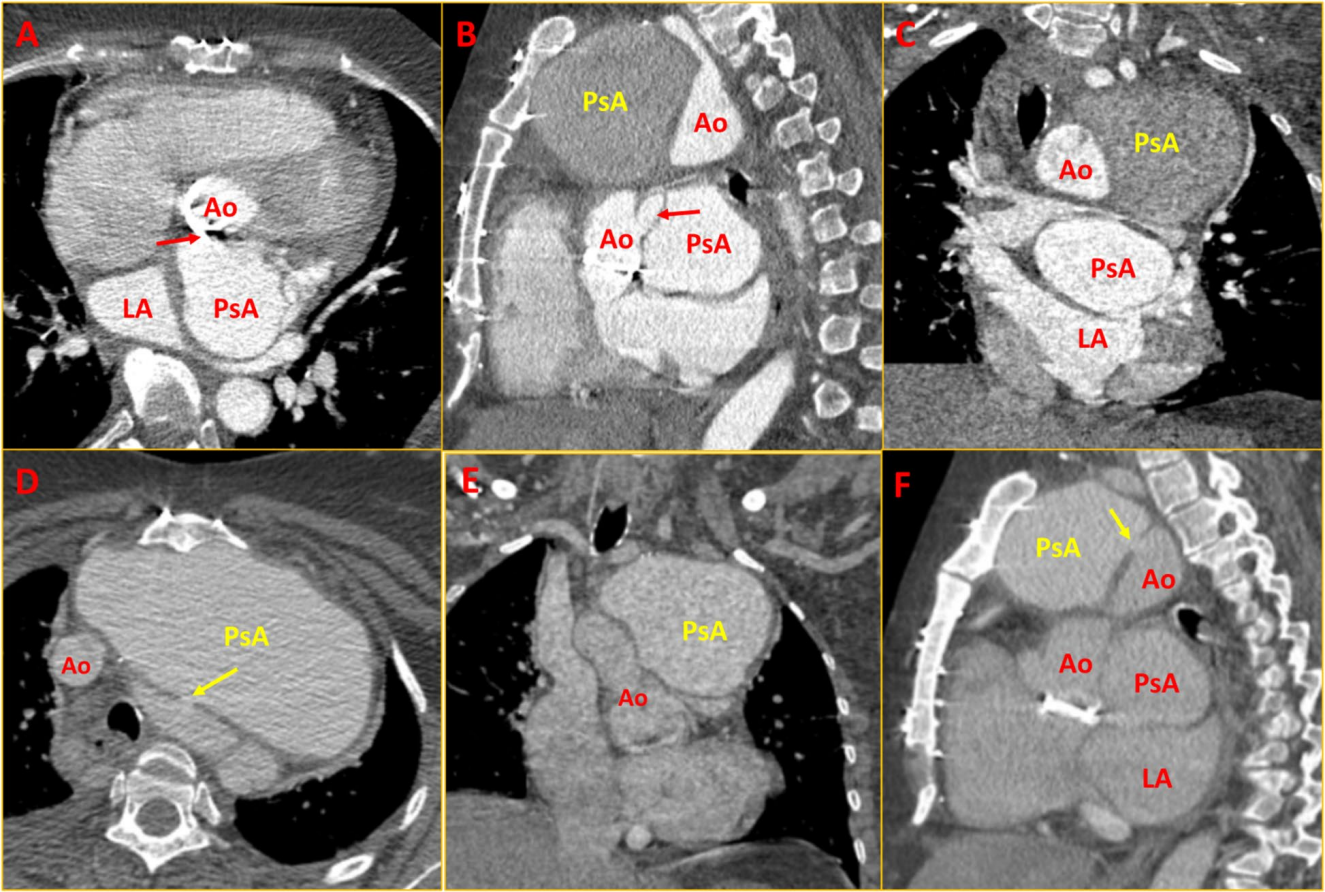
Cardiac CT. **A** Axial CT aortogram showing large PsA arising from aortic root just above the prosthetic valve, compressing the LA (arrow: neck of PsA); **B** Sagittal CT aortogram showing 2 PsAs, one posteriorly from aortic root with good contrast opacification in early phase, second anteriorly from ascending Ao abutting the sternum (note the left coronary artery dilatation [arrow] and LA compression by aortic root PsA); **C** Coronal CT aortogram showing the special relationship of the PsAs with Ao and LA. (D-F) Delayed phase of aortogram in axial (**D**), coronal (**E**), and sagittal (**F**) view showing the above findings (arrow: neck of PsA). CT = computed tomography; PsA = pseudoaneurysm (yellow: at ascending aorta, red: at aortic root); Ao = aorta; LA = left atrium

**Fig. 3:**
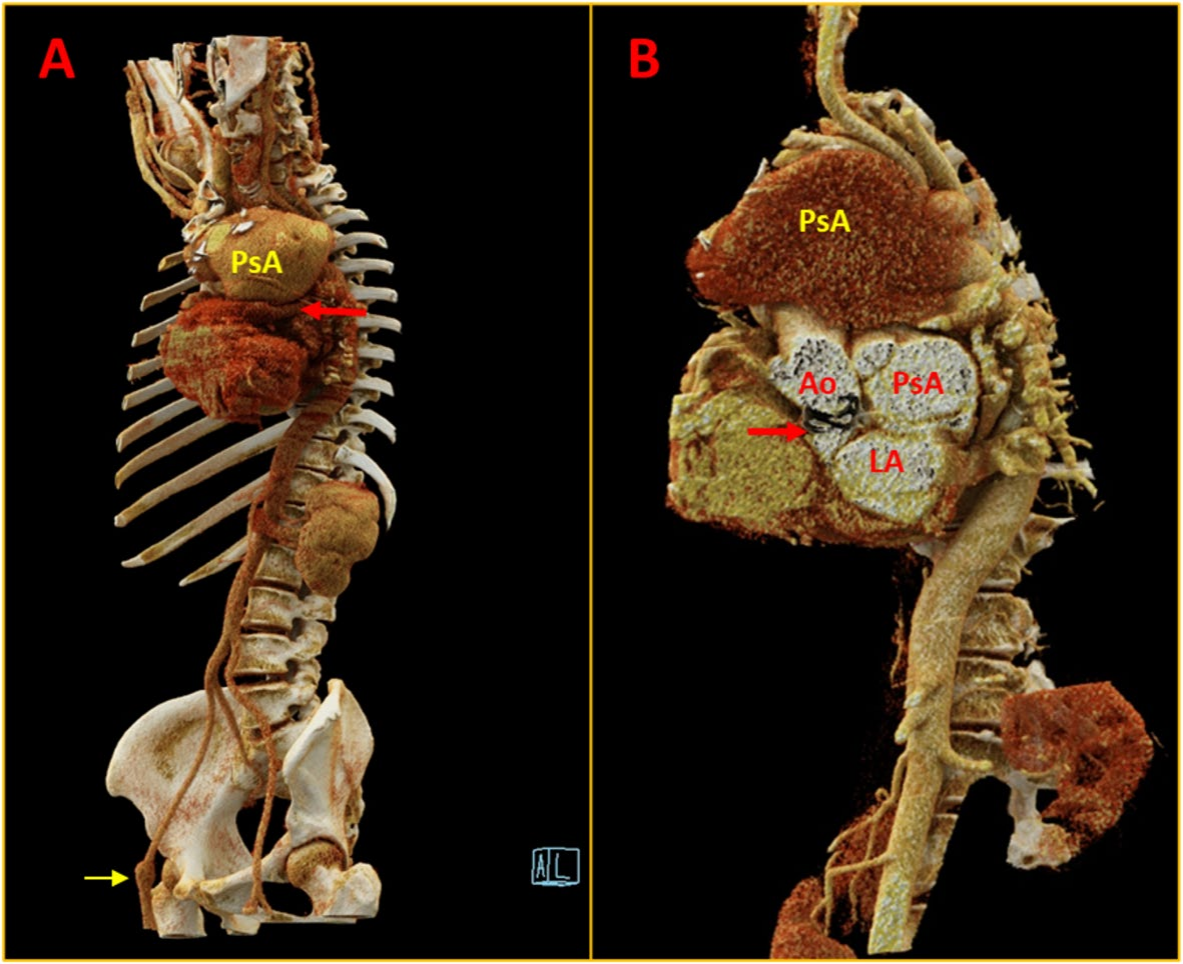
Volume rendered technique clarifying the similar findings. Note the LA compressed by the PsA (red arrow in **A**). Additional aneurysm noted in right femoral artery (yellow arrow: **A**). Aortic prosthetic valve (arrow in **B**), both PsAs and LA relationship depicted in **B**. PsA = pseudoaneurysm (yellow: at ascending aorta, red: at aortic root); Ao = aorta; LA = left atrium

**Fig. 4:**
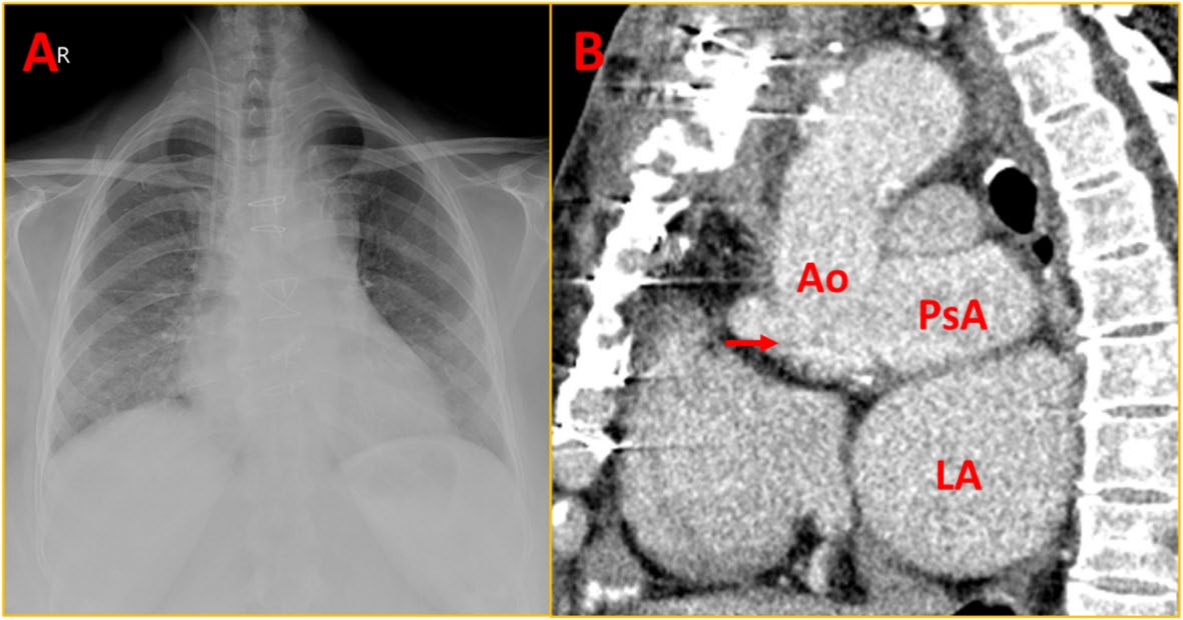
Chest X-ray and Cardiac CT following pseudoaneurysm repair. **A** Chest X-ray showing near complete resolution of mediastinal mass after surgery. **B** Repeat cardiac CT showing persistence of aortic root pseudoaneurysm and resolution of ascending aortic pseudoaneurysm. Arrow indicates the aortic valve level (the beam hardening artifact from prosthetic valve). CT = computed tomography; PsA = pseudoaneurysm at aortic root; Ao = aorta; LA = left atrium

A 48-year-old lady had an ascending aortic aneurysm with severe aortic regurgitation, underwent a Bentall procedure 8 years ago, and was diagnosed as histology-confirmed mucoid vasculopathy (pathologic slides were not available for histologic review). Subsequently, she developed multiple peripheral aneurysms in the left femoral and right popliteal artery for which she underwent surgical repair. Later she developed gradually progressive dyspnoea with dysphagia for 8 months. She was referred in view of a suspected aneurysm in the aortic root and a huge mediastinal shadow on the chest X-ray (Fig. 1A). An echocardiogram revealed a large aortic root pseudoaneurysm adjacent to the left coronary button and a huge ascending aortic pseudoaneurysm with a narrow neck and turbulent flow within (Fig. 1B and C, Supplementary Video 1). Computed tomography (CT) aortogram revealed a huge pseudoaneurysm in ascending aorta measuring 11 cm × 9 cm × 6 cm abutting the sternum with dense adhesions (Figs. 2 and 3, Supplementary Videos 2, 3, 4, 5, 6 and 7). Another pseudoaneurysm was noted at the aortic root adjacent to the left coronary artery measuring 6 cm × 5 cm × 5 cm compressing the left atrium (LA) and oesophagus (Figs. 2 and 3). One peripheral aneurysm was also noted in the right femoral artery (Fig. 3A). During surgery, the pseudoaneurysm was noted to erode into the sternum and ruptured during sternotomy. The bigger aneurysm was repaired. The second aneurysm was not accessible due to dense inflammatory adhesions. A 3-month post-operative chest X-ray showed resolution of mediastinal mass (Fig. 4A). Repeat cardiac CT showed persistence of aortic root pseudoaneurysm and resolution of ascending aortic pseudoaneurysm (Fig. 4B).

A huge pseudoaneurysm following Bentall’s procedure is rare but usually involves coronary buttons [1, 2]. Multiple vascular bed involvement, and large pseudoaneurysms with no clot inside further support our diagnosis of mucoid vasculopathy [3]. However, it is a rare form of vasculopathy, a probable predisposing condition in our case, causing multiple huge pseudoaneurysms being reported first in literature [4].

### Supplementary Information

**Additional file 1: Supplementary Video 1.** 2D echocardiogram in modified left parasternal view.

**Additional file 2: Supplementary Video 2.** Post-contrast cardiac computed tomography axial section.

**Additional file 3: Supplementary Video 3.** Post-contrast cardiac computed tomography coronal section.

**Additional file 4: Supplementary Video 4.** Post-contrast cardiac computed tomography sagittal section.

**Additional file 5: Supplementary Video 5.** Delayed post-contrast cardiac computed tomography axial section.

**Additional file 6: Supplementary Video 6.** Delayed post-contrast cardiac computed tomography coronal section.

**Additional file 7: Supplementary Video 7.** Delayed post-contrast cardiac computed tomography sagittal section.
